# Safety and Tolerability of Antiretrovirals during Pregnancy

**DOI:** 10.1155/2011/867674

**Published:** 2011-04-11

**Authors:** Adriana Weinberg, Jeri Forster-Harwood, Jill Davies, Elizabeth J. McFarland, Jennifer Pappas, Kay Kinzie, Emily Barr, Suzanne Paul, Carol Salbenblatt, Elizabeth Soda, Anna Vazquez, Myron J. Levin

**Affiliations:** ^1^School of Medicine University of Colorado Denver, Research Complex 2, Mail Stop 8604, 12700 E. 19th Avenue, Aurora, CO 80045, USA; ^2^Department of Obstetrics and Gynecology, University of Colorado Hospital, Aurora, CO 80045, USA; ^3^Pediatric Infectious Diseases, The Children's Hospital, Aurora, CO, USA

## Abstract

Combination antiretroviral therapy (CART) dramatically decreases mother-to-child HIV-1 transmission (MTCT), but maternal adverse events are not infrequent. A review of 117 locally followed pregnancies revealed 7 grade ≥3 AEs possibly related to antiretrovirals, including 2 hematologic, 3 hepatic, and 2 obstetric cholestasis cases. A fetal demise was attributed to obstetric cholestasis, but no maternal deaths occurred. The drugs possibly associated with these AE were zidovudine, nelfinavir, lopinavir/ritonavir, and indinavir. AE or intolerability required discontinuation/substitution of nevirapine in 16% of the users, zidovudine in 10%, nelfinavir in 9%, lopinavir/ritonavir in 1%, but epivir and stavudine in none. In conclusion, nevirapine, zidovudine, and nelfinavir had the highest frequency of AE and/or the lowest tolerability during pregnancy. Although nevirapine and nelfinavir are infrequently used in pregnancy at present, zidovudine is included in most MTCT preventative regimens. Our data emphasize the need to revise the treatment recommendations for pregnant women to include safer and better-tolerated drugs.

## 1. Introduction

Combination antiretroviral therapy (CART) has decreased HIV mother-to-child-transmission (MTCT) to <2% in the USA and other countries where ART is readily available [1–5]. To reliably achieve suppression of maternal HIV replication, which is essential for prevention of MTCT, information on the safety and tolerability of drug regimens for HIV-infected pregnant women is critically important. 

Antiretroviral regimens recommended by the WHO for PMTCT (http://www.who.int/hiv/pub/mtct/antiretroviral2010/en/index.html) include zidovudine (AZT) and lamivudine (3TC) with a single dose of nevirapine (NVP) at delivery or AZT/3TC with lopinavir/ritonavir (LPV/RTV), with efavirenz (EFV; only in the 2nd trimester or later) or with abacavir (ABC) for the entire duration of treatment in pregnancy. Overall, the most commonly used nucleoside reverse transcriptase inhibitors (NRTIs) during pregnancy are AZT and 3TC [6]. Some NRTIs are avoided during pregnancy due to their toxicity, such as didanosine (DDI) with stavudine (D4T) [7]. NVP was associated in some studies with a high level of hepatotoxicity in women with CD4 > 200 cells/*μ*L [8, 9]. Although this observation was not confirmed in other studies [10, 11], NVP is currently recommended only as a single dose at delivery or in women with CD4 < 250 cells/*μ*L. Tenofovir (TNV) has not been extensively studied in pregnancy. Its use is limited because of its effect on bone mineralization [12, 13]. 

Although CART for the mother clearly reduces the risk of HIV MTCT, it is not universally used for this purpose because of the high cost of drugs, concern with the safety and tolerability of different classes of antiretrovirals [14–19] and with the potential emergence of drug resistance in mothers who stop CART after delivery [20–23]. In a previous study, we showed that the use of CART by 117 women in our clinic during pregnancy was not associated with drug resistance [24]. This was subsequently confirmed by similar findings in other studies [25]. 

The objective of this study was to evaluate the safety and tolerability of different components of CART during pregnancy.

## 2. Material and Methods

### 2.1. Study Design

This was a retrospective chart review study of CART utilization and adverse events (AEs) in pregnancies managed by the Children's Human Immunodeficiency Program (CHIP) in Denver, Colo between August 1997 and December 2005, as previously described [26]. Basic CART consisted of ≥3 ARV representing ≥2 classes. Hematology, chemistry, and liver function tests were done at 4- to 8-week intervals. For this report, we collected and analyzed AE for pregnancies of at least 16 weeks duration and with at least 2 visits to CHIP and drug substitutions due to grade ≥3 AE or intolerance of ART, defined as inability to tolerate nonlife threatening clinical AEs, such as headache, nausea, diarrhea, or other subjective disorders. AEs were classified as per the Division of AIDS Table for Grading the Severity of Adult and Pediatric Adverse Events, Version 1.0, dated December, 2004 (http://rcc.tech-res.com/safetyandpharmacovigilance/). 

### 2.2. Statistical Analyses

We utilized a two-sided test of hypothesis with a significance level of.05, and it was performed in SAS v9.2. Characteristics are presented as medians with interquartile ranges or number (*N*) and percent in a category, as appropriate. McNemar's test was used to assess whether AZT and D4T were more likely to be added versus discontinued during pregnancy.

## 3. Results

### 3.1. Demographics and HIV-Disease Characteristics

Of 124 pregnancies that met inclusion criteria, complete medical records were available on 117 pregnancies including 12 women with 2 pregnancies (Table 1). Median gestational age at delivery was 38 weeks (interquartile range (IQR) = 37–40 weeks). Of 120 infants (3 twin gestations), 2 were stillborn, 1 died of sepsis at 1 day of life, and 117 survived and were free of HIV.

### 3.2. Antiretrovirals and AEs during Pregnancy

The median duration of continuous therapy during pregnancy was 22 weeks (IQR = 15–35 weeks). Among 115 evaluable women at delivery, 106 (94%) were on CART (≥3 drugs from ≥2 classes); 7 women (4%) were on 2 or 3 NRTI due to lack of tolerance of CART; 1 woman was on AZT monotherapy, and 1 woman refused ART. 

Hematologic, metabolic, hepatic, and pancreatic AEs identified by routine testing were confirmed by repeat testing (Table 2). Grade ≥3 anemia was documented in 2 patients (2%), one of whom also had thrombocytopenia. Both women were receiving AZT when hematologic abnormalities developed. Laboratory values improved after AZT discontinuation suggesting that AZT caused the SAE. Five additional subjects, including 4 on AZT, had grade 1 or 2 hematologic AEs. 

There were 4 grade ≥3 hepatobiliary SAE in 3 of 99 evaluable women (3%), including one with 2 pregnancies. Two women had underlying liver disease caused by hepatitis C virus or hepatic steatosis. The drugs deemed probably responsible for the SAE were ritonavir- (RTV-) boosted saquinavir (SAQ), indinavir (IDV), nelfinavir (NFV), and AZT. Fifteen pregnancies (15%) were complicated by grade 1 or 2 liver function abnormalities, none of which required ARV changes. 

Of 69 women with amylase measurements, 3 (4%) had grade 1 or 2 transient elevations, which normalized without any intervention and, therefore, could not be ascribed to ARV.

Two women were diagnosed with obstetric cholestasis (OC) defined by pruritic rash and elevated bile acids. One of them with chronic hepatitis C infection had a nonviable fetus at the time of OC diagnosis. The other individual without underlying liver disease delivered a healthy infant by Cesarean section. Both individuals were on PI-containing regimens at the time OC was diagnosed: one on NFV and one on lopinavir (LPV) with RTV boost. The patient with underlying hepatitis C had a subsequent pregnancy, during which she received a triple NRTI regimen (AZT/3TC/ABC). She did not develop OC and delivered a healthy, uninfected infant.

Two women developed rashes while on NVP, which improved after NVP discontinuation. There were other clinical AE, including nausea, vomiting, diarrhea, perioral paresthesias, headache, and insomnia that prompted drug discontinuations.

### 3.3. Changes in ART Caused by ARV Intolerance during Pregnancy

Grade ≥3 AE or poor tolerability prompted 17 changes in therapy in 16 out of 117 pregnancies (14%; Table 3). 

There were 11 NRTI substitutions in 114 women receiving NRTI (10%). Ten involved AZT and were due to hematologic SAE, progressive anemia, which in the opinion of the health care provider would reach grade ≥3 before the end of pregnancy, headache, insomnia, or gastric discomfort. Overall, AZT was more likely to be discontinued than added (*P* = .004, McNemar's test). AZT was most commonly substituted by D4T, which was more likely to be added than discontinued (*P* = .02, McNemar's test). The average durations of AZT and D4T therapy during pregnancy were similar at 140 and 111 days, respectively (SD = 83 and 73, resp.). 

NNRTI substitutions occurred in 3 of 21 women (14%) receiving NNRTI other than efavirenz (EFV). All occurred among 19 women on NVP. EFV was substituted in 2 women who inadvertently became pregnant while on EFV. Their infants did not have any gross abnormalities at birth or during follow-up. 

PI substitutions occurred in 8 of 97 pregnancies (8%) with PI-containing CART. LPV/RTV and NFV were the PI most commonly administered. LPV/RTV was used in 28 pregnancies and was substituted or discontinued due to toxicity or lack of tolerability in 2 women (1%). NFV was substituted in 6 (9%) of 64 women. Other PIs, such as saquinavir and indinavir, were less commonly used.

## 4. Discussion

In this study, 8% of pregnancies were complicated by grade ≥3 AE probably or possibly due to ARV which is similar to previous reports [27–29]. SAE probably or possibly related to the use of ARV included 3 hematologic, 4 hepatic, and 2 OC. One woman had multiple SAE during 2 pregnancies. The incidence of ARV-associated SAE and the rate of drug substitutions did not significantly differ across classes of drugs, suggesting that they were equally safe and well tolerated during pregnancy.

Among NRTI, AZT was the most poorly tolerated drug. In the first AZT trial for PMTCT [30], the incidence of AE in mothers receiving AZT monotherapy was similar to that in placebo recipients. However, HIV replication was poorly controlled in that study and may have contributed to the overall incidence of AE. Other studies using combination therapy during pregnancy showed frequent hematologic toxicities in mothers and children who received AZT [27, 31–33]. In our study, although mothers who developed hematologic SAE ascribed to AZT were receiving combination ART at the time of the event, laboratory values improved after AZT substitution, suggesting that AZT was responsible for the AE. Despite its marginal safety and tolerability during pregnancy, AZT is the main drug recommended by USPHS and WHO for PMTCT. This is partly due to the reluctance to substitute a drug with proven efficacy. However, CART has higher or equal efficacy for PMTCT than AZT alone, making it possible to substitute AZT with other drugs to avoid undesirable side effects.

Drugs that could potentially substitute AZT in combination ART for PMTCT are TNV, ABC, and D4T, all of which synergize with 3TC. The Antiretroviral Pregnancy Drug Registry includes data on ≥628 pregnancies for each of these drugs with a rate of congenital birth defects similar to that of the general population [6]. Although these drugs appear nonteratogenic, they face other limitations. TNV interferes with bone formation in experimental animals [13]. In small numbers of reports in humans, results were variable [34, 35], and, therefore, providers tend to avoid TNV in pregnancy. The use of ABC is limited by its potential allergic reactions. This risk can be mitigated by HLA B5701 detection, which defines the likelihood of ABC hypersensitivity [36]. In pregnancy, drug changes have to be quickly implemented, which may not be compatible with the delay required for HLA typing. Finally, D4T has been associated with peripheral neuropathy, lactic acidosis and other metabolic abnormalities including lipodystrophy [37, 38]. Nevertheless, this drug continues to be widely used in resource-limited countries. In our experience, D4T administered for a limited period of time during pregnancy was well tolerated. The average duration of treatment with D4T and AZT were similar in this study, but patients did not have to discontinue D4T during pregnancy. These findings are consistent with other studies that showed a lower rate of substitution of D4T compared to AZT in nonpregnant adults [39, 40]. Furthermore, D4T crosses the placenta and achieves sufficient levels in the fetus for pre-exposure prophylaxis. Although based on a limited number of observations (*N* = 23), our data suggest that D4T may be a viable alternative to AZT during pregnancy. 

NNRTI are uncommonly used during pregnancy other than single-dose NVP at delivery. EFV is contraindicated in the first trimester due its potential teratogenicity [41]. EFV has recently been included in the WHO recommendations for combination ART after ≥14 weeks of gestations, but its use is still limited. Delavirdine and etravirine have been insufficiently studied during pregnancy. NVP, which was widely used for PMTCt in the late 1990s, is currently contraindicated in pregnant women with ≥250 CD4 cells/*μ*L due to potential hepatic and cutaneous toxicity. In this study, NVP was used in 19 women with a median first visit CD4 of 419 (IQR = 205–588). Three (16%) required NVP substitution due to mild or moderate AE.

NFV, the PI most commonly used in this study was poorly tolerated in 6 of 64 women (9%). The second most commonly used PI was LPV/RTV, which was well tolerated by 26 of 28 women. Three women developed 4 episodes of grade ≥3 hepatic AE, 3 of which were associated with PI (NFV, SAQ/RTV and indinavir). In addition, two cases of OC were diagnosed in women receiving PI. The relationship between OC and ARV is not clear. The ARV Registry includes very few episodes of OC, but the registry, which was designed for birth defects, does not systematically collect other AE, which may underestimate their incidence, including OC. OC resulting from a pregnancy-specific accumulation of bile acids is associated with fetal demise [42]. The risk of OC is increased by chronic hepatitis C infection [43], which was present in one of our study women, and by other chronic liver conditions. Otherwise, OC is quite uncommon in Europeans, with an incidence of 0.1 to 2%, but quite common in Chile (9 to 16%), possibly related to the genetic background of the population [44, 45]. PI and other ARV have hepatotoxic potential [11, 46] that may contribute to OC. This hypothesis deserves to be further studied.

In conclusion, the safety and tolerability of CART in pregnancy did not differ by class of ARV, but there were differences among individual drugs. Drugs with the poorest safety and tolerability were AZT, NVP, and NFV. Our findings support the need to devise new CART regimens for PMTCT that will avoid the use of poorly tolerated drugs.

## Figures and Tables

**Table 1:** Demographics and baseline characteristics of HIV infection.

Characteristic	*N* or Median (% or IQR)
Pregnant patients	105
One pregnancy with CHIP	93 (89)
Two pregnancies with CHIP	12 (11)
Maternal age at delivery	30 (26, 34)

Race	
White	72 (68.6)
Black	31 (29.5)
Other	2 (1.9)

Ethnicity	
Hispanic	41 (39)
Not hispanic	64 (61)

HIV risk factors^†^	
IV drug use	12 (11)
Heterosexual sex	98 (92)
Transfusion	5 (5)

Timing of HIV diagnosis	
Prior to first pregnancy at CHIP	70 (67)
During first pregnancy at CHIP	35 (33)
Antiretroviral therapy at the onset of pregnancy	29 (25)
Plasma HIV RNA at first visit	109 pregnancies
Median (quartiles)	2657 (225, 16700)
>400 copies/mL	78 (72)
CD4+ count at first visit	108 pregnancies
Median (quartiles)	450 cells/*μ*L (269, 628)
<200 cells/*μ*L	13 (12%)

^†^Some subjects had multiple risk factors.

**Table 2:** Laboratory adverse events.

Adverse event	*N* tested^#^	*N* (%) with adverse event			
		Grades 4*	Grades 3*	Grades 2*	Grades 1*
Anemia	110	2 (2%)	0	1 (1%)	4 (4%)
Thrombocytopenia	110	0	1 (1%)	0	2 (2%)
Neutropenia	110	0	0	3 (3%)	1 (1%)
Elevated ALT	98	1 (1%)	1 (1%)	4 (4%)	6 (6%)
Elevated AST	99	0	2 (2%)	4 (4%)	8 (8%)
Elevated bilirubin	98	2 (2%)	0	1 (1%)	2 (2%)
Elevated alkaline phosphatase	96	0	0	2 (2%)	17 (18%)
Elevated amylase	69	0	0	2 (3%)	1 (1%)

*AE grades are the maximum observed grade for each pregnancy. Grading was performed according to the Division of AIDS Table for Grading the Severity of Adult and Pediatric Adverse Events, Version 1.0, dated December 2004 (http://rcc.tech-res.com/safetyandpharmacovigilance/).

**Table 3:** Drug substitutions or discontinuations during pregnancy.

Class	Drug	*N* events/*N* pregnancies	Percent (95% confidence interval)
*NRTI*		11/114^#^	10 (5–17)

	AZT	10/99	10 (5–18)
	3TC	0/109	0 (0–3)
	d4T	0/23	0 (0–15)
	ABC	1/8	13 (0–53)
	ddI	0/6	0 (0–46)
	TDF	0/2	0 (0–84)
	FTC	0/1	0 (0–98)

*NNRTI*		3/20^#∗^	15 (3–38)

	NVP	3/19	16 (1–33)
	DLV	0/1	0 (0–98)
	EFV	2/2	n.a.

*PI*		8/97^#^	8 (4–16)

	NFV	6/64	9 (4–19)
	LPV/RTV	2/28	1 (1–24)
	IDV	0/5	0 (0–60)
	IDV/RTV	0/1	0 (0–98)
	SQV	0/2	0 (0–84)
	SQV/RTV	2/9	22 (3–60)

*Excludes EFV substitutions.

^#^In the drug class summary, a pregnancy in which ≥2 drugs from the same class were substituted was counted only once.
